# Alectinib re-challenge in small cell lung cancer transformation after chemotherapy failure in a patient with ALK-positive lung cancer: A case report

**DOI:** 10.1016/j.rmcr.2021.101440

**Published:** 2021-06-01

**Authors:** Akira Yamagata, Toshihide Yokoyama, Yasushi Fukuda, Tadashi Ishida

**Affiliations:** a Department of Respiratory Medicine, Ohara Healthcare Foundation, Kurashiki Central Hospital, 1-1-1 Miwa, Kurashiki, Okayama, 710-8602, Japan; b Department of Respiratory Medicine, Graduate School of Medicine, Kyoto University, 54 Kawahara-cho, Shogoin, Sakyo-ku, Kyoto, 606-8507, Japan

**Keywords:** Non-small cell lung cancer, Small cell transformation, Alectinib, Anaplastic lymphoma kinase

## Abstract

Small cell lung cancer (SCLC) transformation is a rare resistance mechanism to anaplastic lymphoma kinase-tyrosine kinase inhibitors (ALK-TKIs), for which cytotoxic chemotherapy is often initiated. However, no case has been reported so far in which the SCLC component disappeared after chemotherapy and the tumor responded to ALK-TKI treatment again. A 41-year-old, never-smoker man was diagnosed with multiple metastatic lung adenocarcinoma harboring ALK gene rearrangements. After tumor re-growth was treated with alectinib, histological analysis of re-biopsy of the primary lesion showed combined small cell carcinoma, and cytotoxic chemotherapy was administered. After resistance to chemotherapy developed, the third biopsy of the primary lesion showed the original ALK gene rearrangements without the SCLC component. Alectinib was re-administered, and partial response was obtained. Biopsy for ALK-positive lung cancer that progressed after chemotherapy for SCLC transformation might be useful for decision-making regarding the therapeutic strategy.

## Abbreviations

ALK: anaplastic lymphoma kinase
CEA: carcinoembryonic antigen
EGFR: epidermal growth factor receptor
FISH: fluorescence *in situ* hybridization
IHC: immunohistochemistry
NSCLC: non-small cell lung cancer
SCLC: small cell lung cancer
SLX: sialyl-Lewis X-i antigen
TKI: tyrosine kinase inhibitor

## 1 Introduction

Anaplastic lymphoma kinase (ALK) gene rearrangements are present in 3–5% of patients with non-small cell lung cancer (NSCLC). In the past decade, various tyrosine kinase inhibitors (TKIs) showed a dramatic and durable clinical benefit against ALK-positive NSCLC. Nevertheless, drug resistance and recurrent disease still develop in the vast majority of initial responders. The resistance mechanisms in patients with ALK-positive NSCLC comprised ALK gene alterations, such as ALK point mutations and copy-number gains, bypass signaling activation through the activation of other oncogenes, and small cell lung cancer (SCLC) transformation [1,2]. Although standard therapeutic strategies have not yet been established for patients with SCLC transformed from ALK-positive adenocarcinoma, standard therapies for SCLC are recommended for patients with SCLC transformed from epidermal growth factor receptor (EGFR)-mutant adenocarcinoma [3]. However, whether subsequent re-challenge with TKI after chemotherapy provides clinical benefit is not known.

A case of adenocarcinoma harboring ALK gene rearrangements that transformed to SCLC following alectinib treatment is reported. After resistance to cytotoxic chemotherapy developed, the third biopsy of the primary lesion showed the original ALK gene rearrangements without the SCLC component, and alectinib re-challenge showed partial response. This case report shows the importance of repeated biopsy for decision-making regarding therapeutic strategies in ALK-positive lung cancer with SCLC transformation.

## 2 Case description

**Fig. 1:**
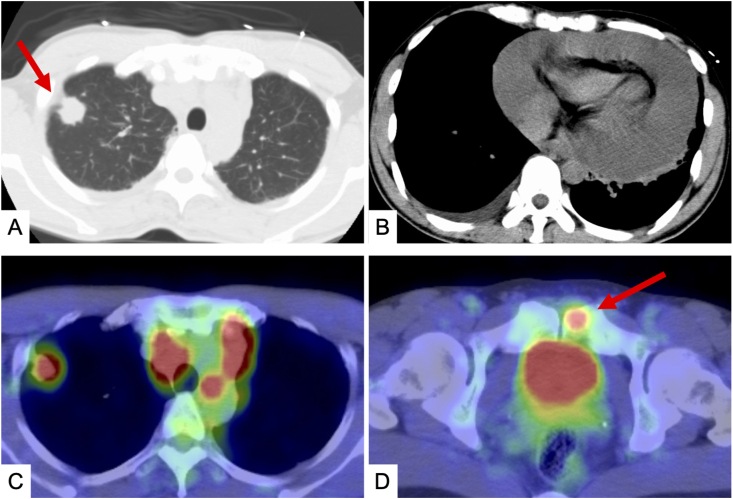
Imaging findings at the time of initial diagnosis A, B) Chest computed tomography scan shows a primary lesion in the right upper lobe and a massive pericardial effusion. C, D) F18 fluorodeoxyglucose positron emission tomography scan shows uptake by the primary lesion, mediastinal lymph nodes, and pelvic bone.

In March 2010, a 41-year old man with no history of smoking underwent a medical examination for epigastralgia. The echocardiogram showed a massive pericardial effusion, and an emergent pericardial puncture was performed. Multiple metastatic lung cancer (cT4N3M1c) was diagnosed by computed tomography and positron emission tomography (Fig. 1). Cytological examination of the pericardial effusion showed adenocarcinoma. The patient subsequently underwent 4 regimens of chemotherapy (cisplatin plus pemetrexed as the first-line treatment, S-1 as the second-line treatment, amrubicin as the third-line treatment, and docetaxel as the fourth-line treatment), but disease progression was observed.

**Fig. 2:**
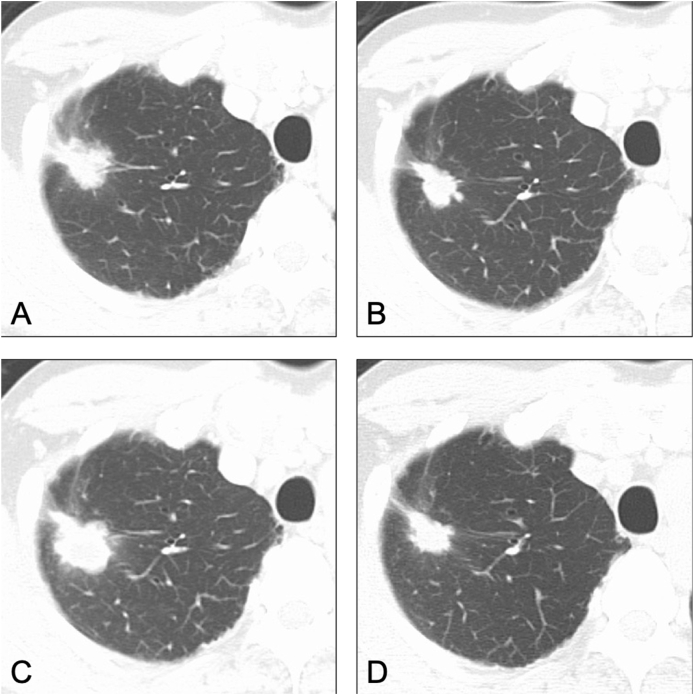
Computed tomography findings A) Progression of the primary lesion after 4 years of alectinib treatment. B) Partial response during cytotoxic chemotherapy for SCLC. C) Progression of the primary lesion after 2 years of cytotoxic chemotherapy for SCLC. D) Partial response after alectinib rechallenge.

In January 2012, a biopsy of the primary lesion in the right upper lobe was performed, and both immunohistochemistry (IHC) and fluorescence *in situ* hybridization (FISH) showed adenocarcinoma with ALK rearrangement. The drug regimen was changed to 300 mg alectinib twice daily, which is the approved dosage in Japan. He eventually achieved a partial response. After 4 years of alectinib treatment, no metastases appeared, but the primary lesion progressed (Fig. 2A).

**Fig. 3:**
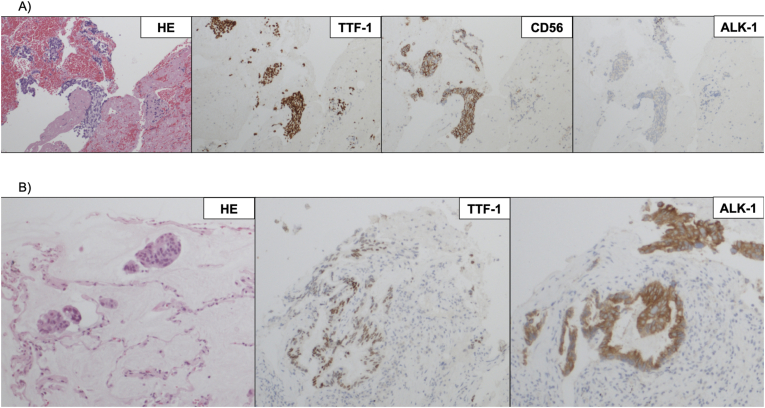
Pathological findings A) The second biopsy sample shows combined small cell carcinoma. SCLC components are positive for CD56 and synaptophysin, and adenocarcinoma components are positive for TTF-1 and ALK-1. B) The third biopsy sample shows only adenocarcinoma without SCLC components. Immunohistopathological analysis shows positive staining for TTF-1 and ALK-1, but negative staining for CD56 and synaptophysin.

**Fig. 4:**
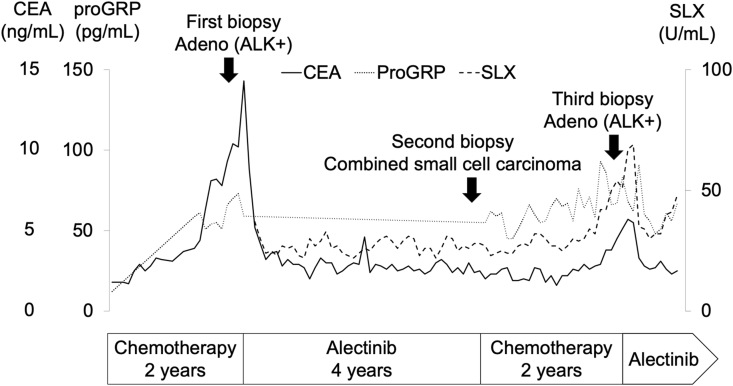
Clinical course during the sequential treatment and tumor marker levels. CEA and SLX levels are re-elevated after disease progression during cytotoxic treatment for SCLC transformation. Alectinib is re-administered after the third biopsy, which results in a partial response and decreased CEA and SLX levels.

Histological analysis based on re-biopsy showed combined small cell carcinoma, in which the SCLC components were CD56 (+), synaptophysin (+), TTF-1 (−), and ALK-1 (−), and the adenocarcinoma components were CD56 (−), synaptophysin (−), TTF-1 (+), and ALK-1 (+) (Fig. 3A). Two regimens of cytotoxic chemotherapy for SCLC (cisplatin plus irinotecan as the sixth-line treatment and amrubicin as the seventh-line treatment) showed a partial response (Fig. 2B), but serum tumor markers such as carcinoembryonic antigen (CEA) and sialyl-Lewis X-i antigen (SLX) increased gradually (Fig. 4). Imaging findings showed progression of the primary lesion and multiple brain metastases (Fig. 2C).

The third biopsy of the primary lesion showed only adenocarcinoma, with CD56 (−), synaptophysin (−), TTF-1 (+), and ALK-1 (+), and disappearance of the SCLC component (Fig. 3B). Alectinib was re-administered, which resulted in a partial response and decreased CEA and SLX levels (Fig. 2, Fig. 4). The tumor response was maintained for 8 months after the rechallenge without cranial irradiation or surgery.

## 3 Discussion

Several cases of EGFR-mutant adenocarcinoma that transformed to SCLC and responded to TKIs again after development of resistance to chemotherapy have been reported [4,5]. To the best of our knowledge, however, this is the first case of successful re-challenge by ALK-TKI in an ALK-positive NSCLC in which the SCLC component disappeared after chemotherapy for SCLC transformation. After transformation, cytotoxic chemotherapy reduced the SCLC component, and the proportion of ALK rearrangement-positive cancer cells increased in the heterogeneous tumors. This may explain why alectinib re-challenge was effective again.

Some reports have shown that the loss of tumor suppressor genes, such as *RB1* and *Tp53*, plays an important role in transformation to SCLC during treatment with ALK-TKIs or EGFR-TKIs [[6], [7], [8]]. In the present case, comprehensive genomic profiling could not be performed due to poor biopsy samples, and the genetic background of SCLC transformation was unknown. However, the absence of a smoking history and tumor progression after long-term molecular targeted therapy suggested that the SCLC components were not originally present, but rather that SCLC transformation was caused by the selective pressure of alectinib treatment.

Serum pro-gastrin-releasing peptide precursor (ProGRP) and neuron-specific enolase (NSE) have been reported to be useful predictive markers for SCLC transformation during the development of resistance to ALK-TKIs [9]. In the present case, there was no correlation between SCLC transformation and ProGRP. On the other hand, CEA and SLX levels were re-elevated after disease progression during cytotoxic treatment for SCLC transformation. No elevation of CEA or SLX was observed when transformation to SCLC was confirmed by the re-biopsy. Thus, these tumor markers may be useful for predicting the majority of the histological subtypes in heterogeneous lung cancers.

In conclusion, the present case is the first to show that re-challenge with ALK-TKIs is effective after chemotherapy failure for SCLC transformation. Repeat biopsy at progression might be useful for decision-making regarding the therapeutic strategy for ALK-positive lung cancer with SCLC transformation.

## Funding

This research did not receive any specific grant from funding agencies in the public, commercial, or not-for-profit sectors.

## Author contributions

AY drafted the manuscript. TY reviewed the manuscript. AY, TY, YF, and TY were responsible for final approval of the version to be submitted.

## Declaration of competing interest

None.
